# Identification and validation of novel prognostic fatty acid metabolic gene signatures in colon adenocarcinoma through systematic approaches

**DOI:** 10.32604/or.2023.043138

**Published:** 2023-12-28

**Authors:** HENG ZHANG, WENJING CHENG, HAIBO ZHAO, WEIDONG CHEN, QIUJIE ZHANG, QING-QING YU

**Affiliations:** 1 Department of Laboratory, Shandong Daizhuang Hospital, Jining, 272051, China; 2 Jining No.1 People’s Hospital, Shandong First Medical University, Jining, 272000, China

**Keywords:** Fatty acid metabolism, Colorectal cancer, Gene signatures, Machine learning

## Abstract

**Background:**

Colorectal cancer (CRC) belongs to the class of significantly malignant tumors found in humans. Recently, dysregulated fatty acid metabolism (FAM) has been a topic of attention due to its modulation in cancer, specifically CRC. However, the regulatory FAM pathways in CRC require comprehensive elucidation.

**Methods:**

The clinical and gene expression data of 175 fatty acid metabolic genes (FAMGs) linked with colon adenocarcinoma (COAD) and normal cornerstone genes were gathered through The Cancer Genome Atlas (TCGA)-COAD corroborating with the Molecular Signature Database v7.2 (MSigDB). Initially, crucial prognostic genes were selected by uni- and multi-variate Cox proportional regression analyses; then, depending upon these identified signature genes and clinical variables, a nomogram was generated. Lastly, to assess tumor immune characteristics, concomitant evaluation of tumor immune evasion/risk scoring were elucidated.

**Results:**

A 8-gene signature, including *ACBD4, ACOX1, CD36, CPT2, ELOVL3, ELOVL6, ENO3*, and *SUCLG2*, was generated, and depending upon this, CRC patients were categorized within high-risk (H-R) and low-risk (L-R) cohorts. Furthermore, risk and age-based nomograms indicated moderate discrimination and good calibration. The data confirmed that the 8-gene model efficiently predicted CRC patients’ prognosis. Moreover, according to the conjoint analysis of tumor immune evasion and the risk scorings, the H-R cohort had an immunosuppressive tumor microenvironment, which caused a substandard prognosis.

**Conclusion:**

This investigation established a FAMGs-based prognostic model with substantially high predictive value, providing the possibility for improved individualized treatment for CRC individuals.

## Introduction

In humans, colorectal cancer (CRC) has peaked incidence rates, annually it accounts for 10% of identified tumor cases together with 9.4% of tumor-linked mortalities. It is the second highly mortal of all 36 human cancers globally, causing death in 900,000 individuals annually [1,2]. Despite the advancements in detection and treatment, it has high mortality due to the absence of specific bio-indices for early identification and prognosis, and so many individuals are clinically diagnosed at advanced stages [3]. Therefore, potential prognostic bio-indices and a comprehensive investigation of CRC molecular mechanisms are crucial for improved prognosis.

Fatty acid metabolism (FAM) involves energy generation/storage, cell membrane growth, together with signaling molecular secretion. Therefore, it has been a hot topic in cancer research [4–6], specifically for CRC [7,8]. Aberrant *de novo* lipid biosynthesis and exogenous fatty acid (FA) uptake cause cancer cells to undergo rapid proliferative and provide essential energetic sourcing source during metabolic stress [9]. Tumor microenvironments’ (TME) FAM alterations, manifested by acidity, high oxidation, hypoxia, and malnutrition due to rapid tumor cells’ proliferation and inadequate angiogenesis, crucially affect cancer [10]. For instance, activated FA oxidation promotes the survival of acute myeloid leukemia cells by remodeling and bone marrow adipocyte lipolysis [11]. In cervical cancer individuals, lymph node metastasis is induced by increased lipolysis and FA synthesis via nuclear factor κB (NF-κB) signaling pathway triggering [12]. Furthermore, elucidating FAM and CRC molecular mechanisms could help identify novel therapeutic targets for effective treatment strategies [13,14].

However, FAM regulatory pathways in CRC have yet to be fully determined. Therefore, elucidating FA metabolic genes (FAMGs) might help explore novel treatment options for CRC. In this investigation, a FAMG signature linked with survival depending upon the colon adenocarcinoma (COAD) clinical and genomic expression datasets acquired through The Cancer Genome Atlas (TCGA) was established. Moreover, other bioinformatics and statistical approaches were carried out to indicate CRC FA and the immunity landscape for novel CRC treatment and drug development.

## Materials and Methods

### Data curation

The colon adenocarcinoma (COAD) clinical and gene expression datasets were gathered from the TCGA-COAD by R package. The Fragments per Kilobase Million (FPKM) value was used to generate the Transcripts per Kilobase Million (TPM) and further subjected to log2 transformation for normalization. Table 1 indicates the clinical history of 446 COAD specimens. Three gene sets related to fatty acid metabolism (Hallmark fatty acid metabolism genes, KEGG fatty acid metabolism pathways, and Reactome fatty acid metabolism genes) were acquired from the Molecular Signature Database v7.2 (MSigDB) [3], and FAMGs were retrieved after over-lapping genes were removed resulting in 309 FAMGs. Then the above FAMGs were corroborated with TCGA with 175 geneomic profiles (Suppl. Table S1). Differentially expressed genes (DEGs) were filtered through “Limma” R software having |log2FC| > 0.5 and false discovery rate (FDR) <0.05 [15]. Gene ontology (GO)/KEGG enrichment tests were performed through Kobas (http://kobas.cbi.pku.edu.cn/).

$$\begin{array}{rl} Risk\;score= & \text{A }gene\times Coef+B\;gene\times Coef+\ldots +\text{X gene}\\ & \times \text{Coef} \end{array}$$

**Table 1:** Clinical characteristics of COAD in the study

Variables	Number (n)	Percentage (%)
Total	446	
Age		
≤65	183	41.0
>65	263	59.0
T stage		
T1	11	2.5
T2	76	17.0
T3	303	67.9
T4	56	12.6
N stage		
N0	265	59.4
N1	102	22.9
N2	79	17.7
M stage		
M0	329	73.8
M1	61	13.7
Unknown	56	12.5
Lymphatic invasion		
No	246	55.2
Yes	159	25.7
Unknown	41	9.1
OS event		
Alive	351	78.7
Dead	95	21.3

### Data processing and risk scoring measurements

The overall survival (OS) was assessed with the help of univariate Cox regression analysis depending upon which similarities/differences in target FAMGs’ microcosmic characters were defined. Then, multivariate Cox regression identified the models’ hub genes to acquire correlation coefficient/s. Identified mRNAs were categorized within protective (0 < HR < 1) and risk (hazard ratio (HR) > 1) types. Finally, a formula for prognostic risk scoring was generated using a linear combination of expression levels compared with corresponding multivariate Cox-formulated regression coefficients. The following formula was utilized for establishing the signature model:

$$\begin{array}{rl} Risk\;score= & \text{A }gene\times Coef+B\;gene\times Coef+\ldots +\text{X gene}\\ & \times \text{Coef} \end{array}$$

where Coef is in Suppl. Table S2.

### PPI network analysis

To further identify core genes signature, a protein-protein interaction (PPI) network was built through the Search Tool for the Retrieval of Interacting Genes (STRING, https://strSuppl.ing-db.org/) website. In addition, Cytoscape was used to visualize the PPI network in Suppl. Fig. S1.

### Construction and prediction model verification

The 446 COAD individuals were categorized within high-risk (H-R) and low-risk (L-R) cohorts depending upon median risk scoring as threshold. Survival curves from the Kaplan-Meier (KM) test were plotted through log-rank test for assessing significance of variations within patients’ survival. Furthermore, receiver operating characteristic (ROC) curves were obtained through R software to measure their area under the curve (AUC) values for individual models to elucidate their efficacy and accuracy. *p* < 0.05 was termed statistically important.

### Nomogram development depending upon fatty acid

#### Metabolic genes and clinical factors

It is believed that clinically a nomogram is a practical tool [16]. Therefore, a nomogram depending upon FAMGs and clinical variables was generated through “rms” R software assistance. Uni-/multi-variate Cox regression determined the corresponding factors linked with prognosis. Consequently, the Akaike information criterion (AIC) selected final nomogram variables through reversed-stepwise variable selection. Lastly, calibration curves were constructed to measure nomograms’ predictive accuracy, concordance index (C-index) was adopted for assessing discriminating ability via Hmisc software (version 4.1.1), and AUC was elucidated to represent its prognostic value by ROC curves.

### Concomitant assessment for immune cell infiltration and the risk scoring within COAD

Tumor mutation burden (TMB) in COAD H-R and L-R cohorts was calculated. The link across risk scoring and the TMB was assessed by Spearman’s correlation assessment. The 5-year survival rate within high-/low- TMB and risk scoring cases, respectively, was determined by the KM test.

### Exploring immune infiltration depending upon eight-gene model

To elucidate the association of FAMGs and tumor immune microenvironment, a box plot indicating the activity of infiltrated immune cells and checkpoint-related gene expression in the two cohorts was constructed via Fluidigm Singular Analysis Toolset 3.5.2 R software. Then, using a violin plot, differences in the Tumor Immune Dysfunction and Rejection (TIDE, http://tide.dfci.harvard.edu/) across H-R and L-R cohorts were elucidated. *p*-value < 0.05 was deemed statistically important.

### Immunohistochemistry (IHC)

Cancer and para-cancer tissues obtained from Jining First People’s Hospital (4–5 μM thick) were utilized for IHC to elucidate the levels of ACOX1, CD36, CPT-2, ELOVL3, and ELOVL6. Briefly, the samples on slides were deparaffinized, rehydrated, blocked, then labeled at 4°C initially with ACOX1 (diluted 1:200, TA0670, Abmart, China), CPT-2, ELOVL3, and ELOVL6 antibody (Abcam, Cambridge, UK) overnight and then with streptavidin-HRP for 40 min. Then the samples were dyed using a 3′-diaminobenzidine (DAB) substrate, counterstained with hematoxylin, and visualized by confocal laser-scanning microscopy (FV1000, Olympus, Japan). The protocols, which included human specimens, were authorized by the Ethical Board of Jining First People’s Hospital [No. 2021LSYD (097) H].

## Results

### Overview of fatty acid metabolic genes in COAD

**Figure 1:**
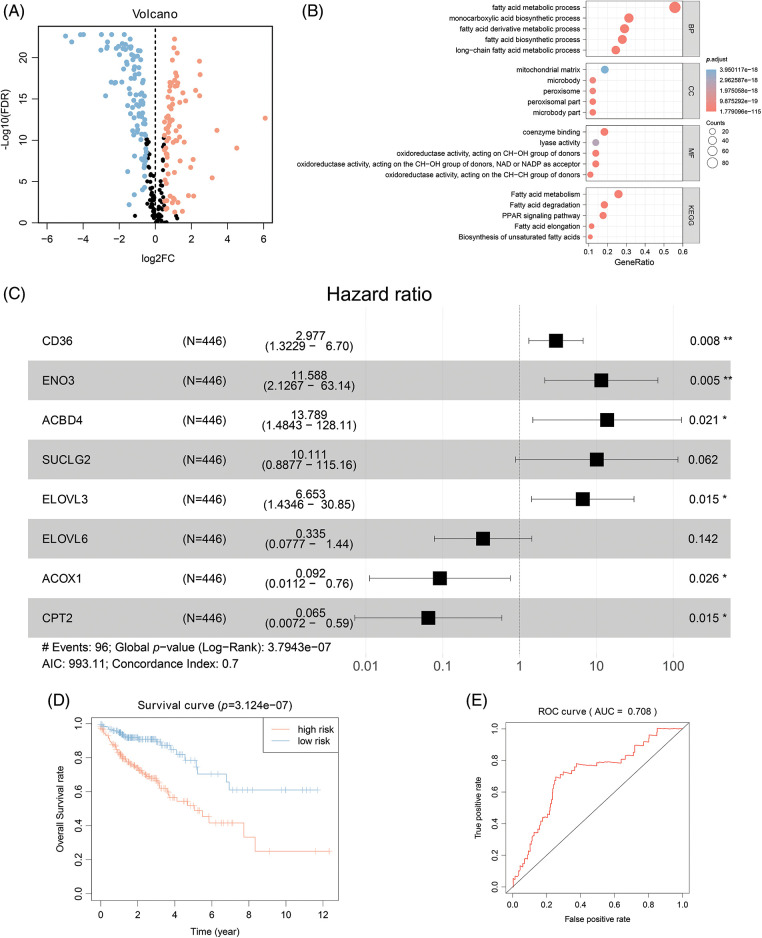
Eight-gene Signatures model to predict COAD outcome. (A) Differentially expressed FARGs in colon cancer (The blue pots represent the downregulation of gene, and the red pots represent the upregulation of gene). (B) The GO enrichment analysis of FARG. (C) Multivariate COX regression analysis for assessing the associations of FARGs with OS in COAD. (D) Kaplan-Meier survival analysis of the differential prognosis across H-R and L-R cohorts in COAD. *p < 0.05, **p < 0.01.

Initially, the differentially expressed FAMGs in the COAD cohort of TCGA were identified, then after intersecting the data of two cohorts, the overlapping FAMGs were selected for subsequent analyses (Suppl. Table S1). Furthermore, the DEGs were also selected by comparing COAD and normal samples at a cutoff of |log2FC| > 0.5 and FDR < 0.05, determining that 175 FMGs were substantially different in COAD patients (Fig. 1A), including 97 genes are upregulated and 78 genes are downregulated. Moreover, GO enrichments analysis was applied to comprehensively elucidate FAMG pathways in COAD, indicating that FAMGs were primarily linked with the coenzyme binding, fatty mitochondrial matrix, and FAM and degradation (Fig. 1B).

### Identifying survival-related FAMGs in COAD

To elucidate the possible association of FAMGs with OS in COAD, univariate Cox analysis was carried out, and survival-related FAMGs (*ACADL, ACBD4, ACOX1, ALAD, CD36, CIDEA, CPT2, ELOVL3, ELOVL6, ACOT11, ENO2, ENO3, HADH, MORC2, SUCLG2*) were identified (*p* < 0.05) (Suppl. Table S3 include HR, gene symbol, 95% CI, and *p*-value of survival-related FAMGs). Next, to identify the most significant FAMSs, the multivariate Cox regression test was utilized to assess their correlation and patient survival (Fig. 1C). FAMGs were associated with stepwise elimination modeling and obtaining their corresponding coefficients. Eight genes (*ACBD4, ACOX1, CD36, CPT2, ELOVL3, ELOVL6, ENO2, and SUCLG2*) were identified (Suppl. Table S2) and categorized into risky types (*ACBD4, CD36, ELOVL3, ENO2, SUCLG2*), with HR > 1 indicating substandard prognosis, and the protective type (*ACOX1, CPT2, and ELOVL6*), with HR < 1 depicting better prognosis (Fig. 1C).

### Construction and validation of eight-gene signature for predicting survival of COAD

**Figure 2:**
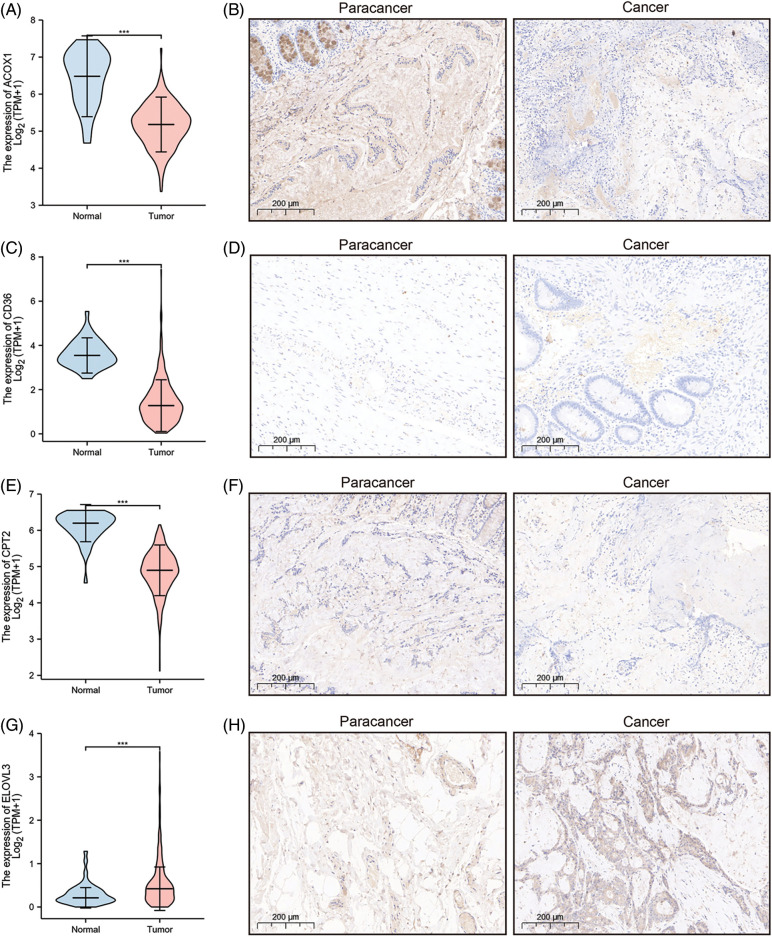

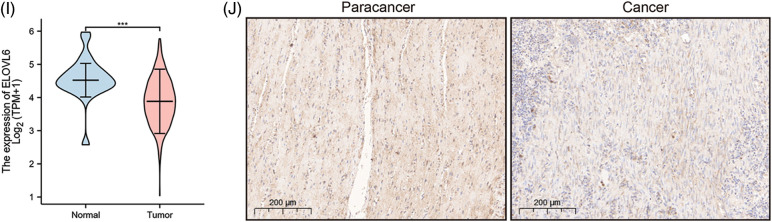
The differential analysis and IHC (5×) of the level of ACOX1 (A and B), CD36 (C and D), CPT-2 (E and F), ELOVL3 (G and H) and ELOVL6 (I and J). ***p < 0.001.

A prognostic model was established considering multivariate Cox regression data for elucidating association for eight FAMGs with COAD prognosis. The derived prognostic risk scoring formula revealed: Risk scoring = *CD36* mRNA expression level × 1.090824766 + *ENO3* mRNA expression level × 2.449937292 + *ACBD4* mRNA expression level × 2.623886937 + *SUCLG2* mRNA expression level × 2.313619642 + *ELOVL3* mRNA expression level × 1.895084335 + *ELOVL6* mRNA expression level × (−1.093983349) + *ACOX1* mRNA expression level × (−2.387977395) + *CPT2* mRNA expression level × (−2.73500507). The KM survival test indicated that the model efficiently predicted COAD prognosis across L-R and H-R cohorts, where the L-R cohort showed enhanced survival rates in comparison to H-R cohort (*p* < 0.0001) (Fig. 1D). Consequently, with ROC curves, models’ predictive value was verified, and the AUC of the ROC was almost 0.7, indicating an adequate predictive ability (Fig. 1E). Furthermore, differential analysis and IHC showed that the level of core gene signatures including ACOX1 (Figs. 2A and 2B), CD36 (Figs. 2C and 2D), CPT-2 (Figs. 2E and 2F), ELOVL3 (Figs. 2G and 2H), and ELOVL6 (Figs. 2I and 2J) were consistent with the above results.

### The integrated risk scoring and clinical parameters analysis of COAD

**Figure 3:**
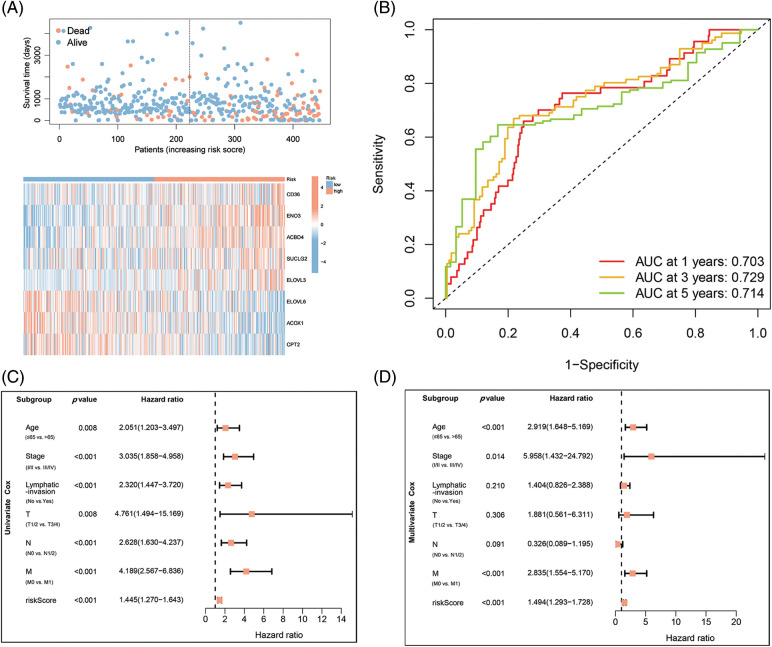
Identification of independent prognostic parameters in COAD. (A) Distributions for OS, risk scoring, and transcriptomic expression profiles. (B) AUC of time-dependent ROC curves validated risk scorings’ prognostic accuracy in COAD. (C and D) Uni- and multi-variate Cox regression assessments for clinic-pathological variables (age, gender, lymphatic invasion, and stage (T, N, and M)) and OS risk scoring in COAD.

The association of COAD patients’ survival status with survival times was ranked by risk scorings, and the heatmap indicated expression data of eight genes (Fig. 3A). The expression of risky types (*ACBD4, CD36, ELOVL3, ENO2*) was enhanced in the H-R cohort than in the L-R cohort. Conversely, the expression of protective types (*ACOX1, CPT2, ELOVL6, SUCLG2*) was reduced in the H-R cohort than in the L-R cohort, implying that the eight-gene model accurately predicted prognosis and their possible effect on tumor incidence and growth. Overall, 1, 3, and 5 ROC curves verified prognostic model’s predictive efficiency; the ROC AUCs were all almost 0.7, indicating an adequate predictive ability (Fig. 3B).

### Constructing FAMGs-clinical nomogram to predict COAD outcomes

**Figure 4:**
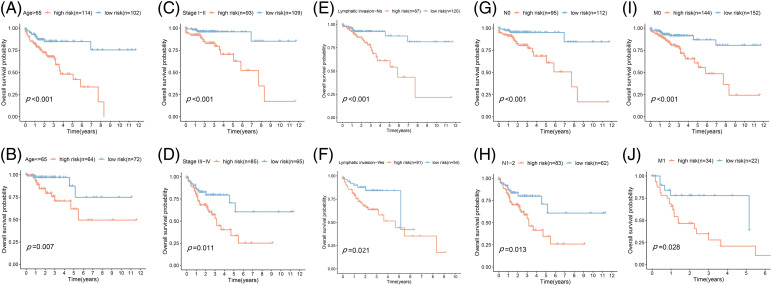
Prognostic validation of FA subtypes in COAD of each clinical parameter. Kaplan-Meier test for OS linked with age (A and B), stage (C and D), lymphatic invasion (E and F), N-stage (G and H), and M-stage (I and J) across H-R and L-R cohorts in COAD.

**Figure 5:**
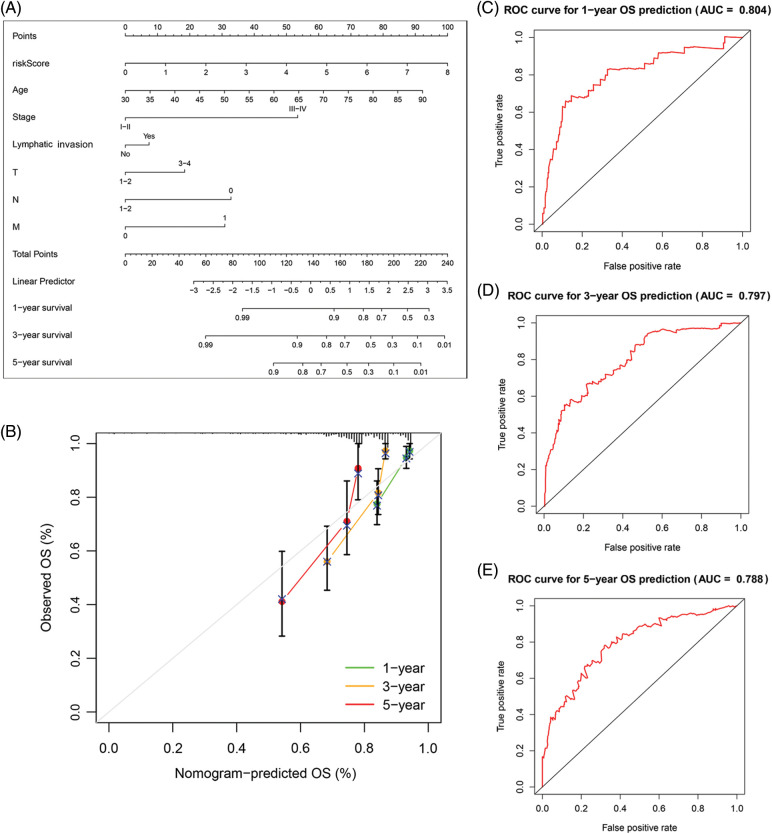
The predictive value of FARGs risk scoring in combination with clinical pathological characteristics in OS of patients from TCGA. (A) Nomogram predicts the OS of cohorts acquired from TCGA. (B) The nomograms’ calibration plots. X-axis = nomogram-predicted survival, and Y-axis = actual survival. (C–E) AUC of time-dependent ROC curves verified the predictive nomogram models’ prognostic accuracy.

Based on the data from eight gene signatures, a nomogram was prepared to for additionally predicting individual case survival rate according to FAMGs and clinical factors. First, the risk scorings and other clinical variables as covariates were assessed via uni- and multi-variate Cox regression models. Univariate Cox data indicated that age, lymphatic invasion, stage (T, N, and M), and risk were OS-linked factors (Fig. 3C). Multivariate Cox analysis-based stepwise forward selection was performed by applying AIC (Fig. 3D), and all the above variables were selected for subsequently developing nomograms. Additionally, The KM survival curves indicated better survival rates in the L-R cohort than the H-R cohort in each sub-cohort (age, lymphatic invasion, stage) (Fig. 4), suggesting that risk scoring-based eight-gene signature is a good prognostic indicator. The nomogram indicated that increased total scoring correlated with reduced survival timeframes (Fig. 5A). A good association was observed across predicted and actual values, validated by the nomograms’ calibration curve for 1-, 3-, or 5-years’ survival probability (Fig. 5B). Then 1-, 3-, or 5-years’ROC curves were applied for validating prognostic model prediction ability; the ROC AUCs = 0.7, indicating an adequate predictive ability (Figs. 5C–5E).

### Conjoint analysis of the tumor mutation burden in COAD

**Figure 6:**
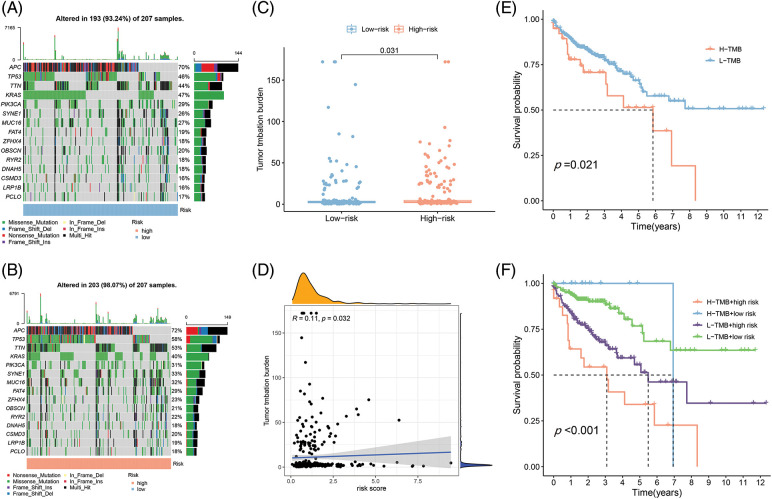
The differences of TMB in the High-Risk and Low-Risk cohorts of COAD. (A) The TMB in L-R cohorts of COAD. (B) The TMB in H-R cohorts of COAD. (C) The different TMB in the H-R and L-R cohorts of COAD. (D) The TMB was positively correlated with the risk scoring in COAD. (E and F) The different OS of the COAD of High- or Low-TMB (E) or with H-R and L-R scoring (F).

Different immune infiltration levels may explain why individuals with histological types of cancer have diverse clinical outcomes [17]. The TMB is a marker for identifying cancer cases that might gain through immunotherapy and can predict outcomes of immune checkpoint inhibitors [18]. As Figs. 6A and 6B depict, the top 10 gene types (APC, TP53, TTN, KRAS, PIK3CA, SYNE1, MUC16, FAT4, ZFHX4, OBSCIN, RYR2, DNAH5, SMD3, LRP1B, PCLO) contributing to TMB are same in the L-R and H-R cohorts and the difference of expression of the above genes was statistically important between the two groups (Fig. 6C). Furthermore, according to the correlation analysis, TMB had positive association to risk scoring (R = 0.11, *p* = 0.032) (Fig. 6D). KM survival analysis revealed that COAD individuals with high TMB have a substandard prognosis (Fig. 6E). Considering the combined impact of TMB and risk scoring on prognosis, the L-R cohort had a better survival rate than the H-R cohort in the low or high TMB cohort (Fig. 6F).

### The landscape of tumor-immune microenvironment in COAD

**Figure 7:**
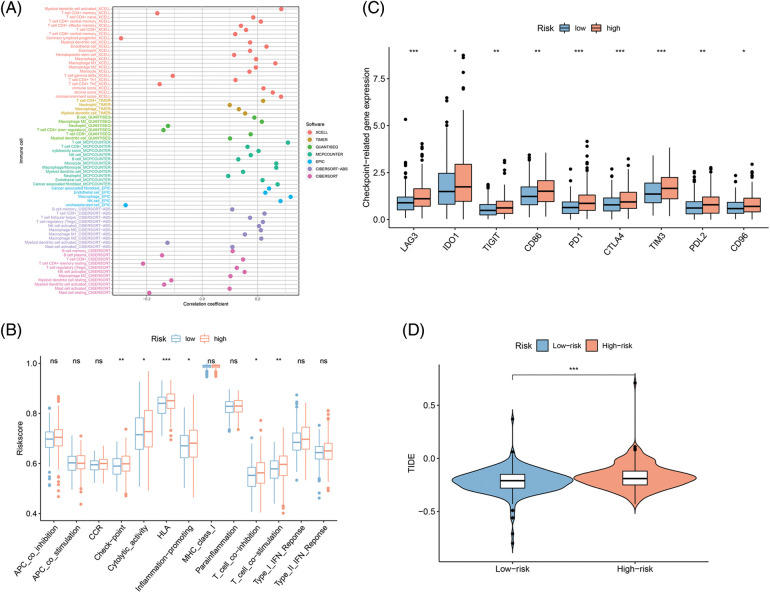
Fatty acid metabolism model in the Tumor immune microenvironment and immunotherapy role in COAD. (A) The association of 24 immune cells with immune infiltration. (B) The known function associated with immunity regulation difference between COAD with H-R and L-R scoring. (C and D) The different checkpoint-related gene expression (C) and TIDE (D) in H-R and L-R COAD cohorts. *p < 0.05, **p < 0.01, ***p < 0.001.

Since immunotherapy for treating CRC showed success [19,20], the correlations of FAMs with immune infiltration levels were also assessed to understand the associated mechanism by which the eight-gene signature influences colon cancer prognosis. Initially, the link between 22 immune cells and immune infiltration was observed (Fig. 7A), which indicated the involvement of T cells, B cells, and macrophages was observed for subsequent analyses. Checkpoint, cytolytic activity, HLA, and inflammation were stimulated in the H-R cohort, indicating that this cohort had immune escaping tumor progress by ssGSEA (Fig. 7B). Novel tumor immune response molecules, including immune checkpoints, have been assessed in the preclinical or clinical trials for cancer development [21–23]. Therefore, expression profiles for nine candidate immune checkpoints were compared across L-R/H-R cohorts, and it was revealed that the expression of targets encompassing *LAG3, IDO1, TIGIT, CD86, PD-1, CTLA4, TIM3, PD-L1*, and *CD96* was markedly increased in the H-R cohort (Fig. 7C). The data indicated that H-R COAD patients with worse prognoses might have an enhanced immune escape to the above checkpoints targeting therapies. TIDE, a computational model, simulates two primary tumor immune evasion mechanisms that can provide predictive immunotherapy outcomes. Elevated TIDE may predict non-responders who have suppressor cells to block T-cell infiltration. According to the violin plot, the TIDE level was higher in the H-R cohort in comparison to L-R cohort (*p* < 0.05), indicating that H-R cohort had increased immune escape resulting in substandard prognosis (Fig. 7D).

## Discussion

CRC is malignant cancer with enhanced mortality and prevalence [23]. Therefore, elucidating its primary molecular pathways and reliable prognostic bio-indices are urgently needed for improved prognosis and individualized therapy. After comprehensive metabolic reprogramming research, scientists gradually realized that FAM is important in CRC [7,12,24,25]. Although the literature mostly focuses on the function of a single FAM regulator, the integrated activity of multiple FAMGs still needs to be discovered. Elucidating the activity of distinct FAM patterns in CRC could allow for determining its association with CRC progression and guide an efficient therapeutic strategy.

As per our knowledge, this is the 1st investigation exploring the link between FAMGs and CRC prognosis. Here, a prognostic risk scoring model was generated considering eight differentially expressed FAMGs identified from the normal and CRC tissues acquired from GEO and TCGA, respectively, via univariate Cox regression analysis. The model predicted CRC patients’ OS to determine the function of these 8 genes. There were differences in L-R and H-R scoring CRC cohorts’ survival rates. The model was a separate prognostic factor in multivariable analysis. Additionally, stage (T, N, and M), lymphatic invasion, age, and risk were discovered as stand-alone OS-linked variables that—when used within such a nomogram—suggested that this nomogram could be applied clinically for diagnosis and CRC treatment. These data indicated that an eight-gene signature efficiently predicted the prognosis of CRC tumor immune evasion and may help guide future clinical treatment strategies.

Through machine learning algorithms and Cox regression, eight OS-related hub FAMGs were identified including ACBD4, ACOX1, CD36, CPT2, ELOVL3, ELOVL6, ENO3, and SUCLG2. The literature shows that these eight hub genes are substantially linked with tumor progression, invasion, and metastasis. ABCD family was upregulated within hepatocellular carcinoma (HCC) [26] and prostate cancer [27], which is involved in cancer progress via peroxisomal enzymes [28]. However, Liao et al. indicated that ACBD4 could serve as a target p53 gene, dysregulated in CRC cells by inauhzin, thereby inhibiting tumors [29]. Acyl-CoA oxidase 1 (ACOX1) represses CRC growth through modulating palmitic acid in β-catenin activation and cancer progression. Therefore, inhibiting ACOX1 dephosphorylation through DUSP14 or β-catenin palmitoylation can be used as CRC therapy [30]. CD36 has diverse effects, including anti-tumor and tumor-promoting (such as CRC tumorigenesis) activities. For instance, CD36 inhibits β-catenin/c-myc signaling by stimulating the proteasome-dependent ubiquitination of GPC4, subsequently suppressing the downstream aerobic glycolysis and tumorigenesis in CRC [31]. However, inhibition of FA synthase enhances *CD36* expression, increasing tumor growth in various CRC models [32]. One of the CRC prognostic markers is CPT2, and its downregulation promotes tumor growth and represses apoptosis via the p53 pathway [33]. Increased ENO3 levels predict substandard prognosis and increase cell glycolysis, promoting CRC aggressiveness [34]. BRG1, a chromatin remodeling protein, activates *ELOVL3* transcription, thereby stimulating migrative/invasive properties of prostate cancer cells [35]. Elovl6 enhances liver cancer oncogenic function and is linked with substandard HCC prognosis [36]. A significant link between *SUCLG2* gene rs35494829 and colon cancer was observed [ORs (95% CIs) per increment for minor allele, 0.82 (0.74–0.92)] [37]. Overall, the novel FAMG signatures identified in this research could serve as efficient CRC prognostic markers, and elucidating their CRC progression pathways is worth investigating.

The 8 FAMG signatures could serve as efficient therapeutic/prognostic CRC targets, while additional elucidation of their relationships with CRC would be recommended. Following constructing a predictive risk model depending upon these 8 FAMGs, with the help of the ROC curve, CRC patients were categorized into H-R and L-R cohorts with an optimistic model-based definition. Consequently, risk scoring was integrated with CRC clinical variables (age, sex, stage, and grade), which revealed that risk and stage were independent prognostic variables, suggesting the models’ practicability. Next, the hub FAMG-based risk scoring and stage were utilized for preparing a predictive tumor model together with a nomogram. Such nomograms’ accuracy for assessing individual-case prognosis was elucidated via ROC curve, C index, and calibration plot analysis. The four validation assays indicated the nomograms’ practicability at different levels. The model might identify CRC individuals and substandard prognoses, assisting in timely interventions. Recently, increased TMB has been linked to curative impact of immune checkpoint inhibitor therapy [38]. FA levels within micro-environment influence infiltrating immune cells’ activity/phenotype [39,40]. The literature suggests that FAM modulation might improve the effect of immunotherapy in cancer individuals. This investigation observed 12 different infiltrating immune cells in H-R and L-R COAD cohorts, which might improve individualized immunotherapy and treatment effects.

As per our understanding, this is the 1st investigation that comprehensively analyzed FAMGs linked with the prognosis of CRC patients’ immunity using the public database. However, this investigation had certain limitations, (1) It is depending upon the dataset acquired through public databases, that may have contributed towards selection bias. Consequently, a large-scale, multicenter investigation is required to validate the clinical implementation of the established model. (2) The research failed to specify the mechanism of FAMGs which affected immune TME escape; that is, because of heterogeneity and complexity displayed by immune micro-environment, immune cells’ infiltration might have resulted from different factors (including different cytokines, chemokines, and chemoresistance) and these factors were not considered. (3) The comprehensive analysis of the modulatory effects of various stress-response pathways on important transcription factors was also not assessed. This investigation only provided preliminary data on the relationship of FAMG level with tumor immune escape. Specific mechanisms of action and modulatory relationships require more studies. (4) Some essential prognostic factors, including chemoradiotherapy and the degree of inflammation in the adjacent tissue, were unavailable within such public database. Therefore, the effect by such parameters upon the nomogram is undetermined.

## Conclusion

Overall, this was the first research to construct and validate a prognostic 8-FAMGs signature depending upon the expression of *ACBD4, ACOX1, CD36, CPT2, ELOVL3, ELOVL6, ENO2, and SUCLG2* for CRC individuals under strict standards. The signature had robust prediction efficiency for CRC prognosis. Furthermore, the infiltrating immune cell variability between H-R and L-R CRC cohorts was assessed to provide a synergistic effect for FAM-targeted treatments and immunotherapy. The data suggested that the novel FA-related gene signature could help develop individualized treatments and targeted drugs and enhance OS within CRC cases.

## Supplementary Materials

Figure S1 The protein-protein interaction (PPI) network of core gene signatures.

## Data Availability

The original contributions presented in the study are included in the article/Supplementary Materials. Further inquiries can be directed to the corresponding authors.

## Abbreviations

CRC: Colorectal cancer
FAM: Fatty acid metabolism
FA: Fatty acid
TME: Tumor microenvironments
FAMGs: FA metabolic genes
COAD: Colon adenocarcinoma
TCGA: The Cancer Genome Atlas
FDR: False discovery rate
GO: Gene ontology
KEGG: Kyoto Encyclopedia of Genes and Genomes
OS: Overall survival
KM: Kaplan-Meier
ROC: Receiver operating characteristic
AUC: Area under the curve
AIC: Akaike information criterion
TMB: Tumor mutation burden
TIDE: Tumor Immune Dysfunction and Rejection

## Supplementary Materials

The supplementary material is available online at https://doi.org/10.32604/or.2023.043138.
