# Supplementary figures and images for: Identification and validation of novel prognostic fatty acid metabolic gene signatures in colon adenocarcinoma through systematic approaches
Source: Oncol Res. 2023 Dec 28;32(2):297–308. doi: 10.32604/or.2023.043138 (PMC10765130; doi:10.32604/or.2023.043138)

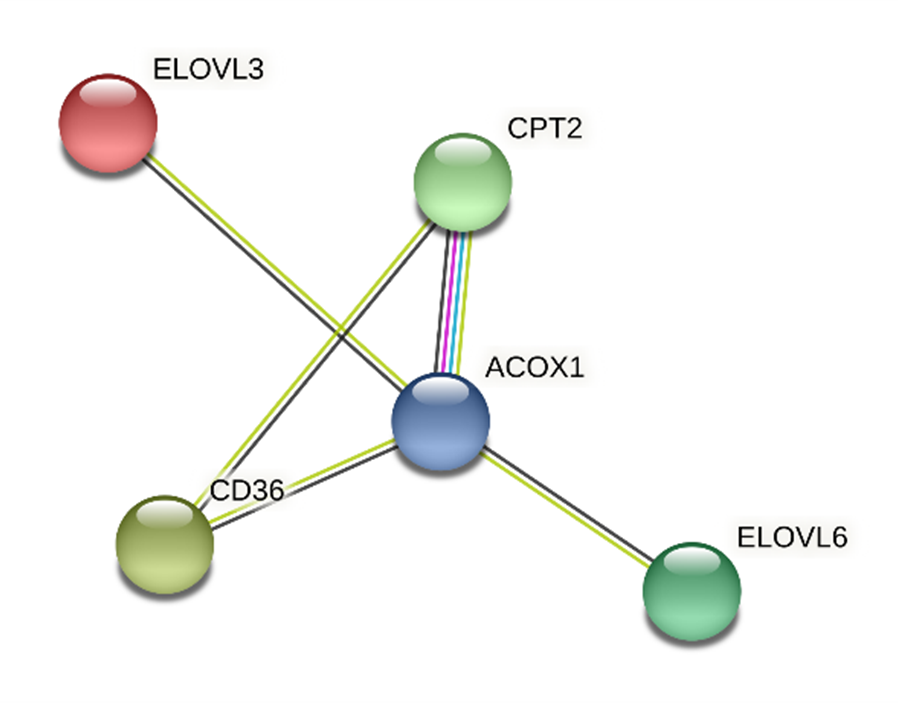

Supplement: Figure S1 [file OncolRes-32-43138-s001.tif]
