# Supplementary material for: Identification and validation of novel prognostic fatty acid metabolic gene signatures in colon adenocarcinoma through systematic approaches
Source: Oncol Res. 2023 Dec 28;32(2):297–308. doi: 10.32604/or.2023.043138 (PMC10765130; doi:10.32604/or.2023.043138)
Supplement: Supplementary file 2 [file OncolRes-32-43138-s001.docx]

**SUPPLEMENTARY TABLE 1**

**Fatty acid metabolism-related differentially expressed genes in COAD**

| **Gene** | **conMean** | **treatMean** | **log2FC** | ***p*-value** | **FDR** |
| --- | --- | --- | --- | --- | --- |
| FADS2 | 1.31175868 | 5.087042461 | 1.955324799 | 1.21E-07 | 1.96E-07 |
| SDHA | 33.41846976 | 20.71662124 | -0.689856947 | 8.06E-16 | 2.71E-15 |
| ACSBG1 | 0.080917123 | 0.053305491 | -0.602160883 | 5.59E-08 | 9.23E-08 |
| PTGES2 | 21.56858073 | 35.49812546 | 0.718811596 | 4.85E-12 | 1.11E-11 |
| CBR1 | 36.44951951 | 24.1180655 | -0.595785603 | 3.59E-05 | 5.19E-05 |
| HPGD | 38.75770171 | 6.168520605 | -2.651486588 | 4.63E-24 | 1.25E-22 |
| CD1D | 3.368119612 | 1.137439197 | -1.566153948 | 8.76E-21 | 6.37E-20 |
| PTGES | 5.135781171 | 10.22710368 | 0.993741994 | 0.000783494 | 0.001028096 |
| AADAT | 1.207334227 | 3.045302032 | 1.334760208 | 8.67E-13 | 2.14E-12 |
| ACAA2 | 70.02742268 | 23.17981389 | -1.595051008 | 4.55E-21 | 3.40E-20 |
| MAOA | 81.82258707 | 23.02434865 | -1.82933881 | 8.18E-24 | 2.00E-22 |
| PRDX6 | 195.9673373 | 78.8850729 | -1.312788979 | 4.40E-22 | 4.29E-21 |
| CD36 | 4.426706927 | 0.988425565 | -2.163029632 | 5.17E-23 | 7.42E-22 |
| ACADVL | 83.69764317 | 56.26949981 | -0.57283386 | 4.17E-13 | 1.06E-12 |
| PECR | 6.031217244 | 3.559977299 | -0.76058116 | 4.81E-14 | 1.36E-13 |
| GPX1 | 91.28805098 | 159.6635585 | 0.806537132 | 1.49E-13 | 3.93E-13 |
| ENO3 | 0.553748493 | 1.099209065 | 0.989163035 | 9.02E-11 | 1.81E-10 |
| ACBD4 | 4.372355854 | 3.086183316 | -0.502587063 | 1.43E-09 | 2.68E-09 |
| CEL | 0.236457205 | 16.03396828 | 6.08340861 | 7.75E-14 | 2.13E-13 |
| CYP4F8 | 0.008953101 | 0.079294616 | 3.146763503 | 2.37E-06 | 3.58E-06 |
| ACBD6 | 2.571598829 | 5.374561837 | 1.063481544 | 1.68E-24 | 5.65E-23 |
| MORC2 | 4.413073049 | 8.489370486 | 0.943873949 | 2.48E-21 | 1.96E-20 |
| CYP2C9 | 0.682349312 | 0.303935576 | -1.166744924 | 0.00028638 | 0.000395058 |
| ADH1A | 0.107122795 | 0.026974997 | -1.989570831 | 4.47E-22 | 4.29E-21 |
| DPEP3 | 0.214518658 | 0.044800538 | -2.259515178 | 2.59E-18 | 1.20E-17 |
| ALOXE3 | 0.008930044 | 0.094924843 | 3.410046601 | 2.67E-12 | 6.35E-12 |
| EHHADH | 9.407654171 | 4.476751906 | -1.071382658 | 8.27E-17 | 3.22E-16 |
| ACACA | 3.474399683 | 6.738458195 | 0.955654804 | 1.36E-15 | 4.42E-15 |
| ALDH1A1 | 52.48914537 | 30.13579422 | -0.80054102 | 4.00E-11 | 8.40E-11 |
| ACAA1 | 14.12476339 | 7.934170241 | -0.832075442 | 6.93E-17 | 2.74E-16 |
| CA4 | 185.2232925 | 5.892073074 | -4.974346401 | 5.44E-25 | 2.44E-23 |
| OLAH | 0.009480618 | 0.0412559 | 2.121547453 | 0.000421969 | 0.000573282 |
| PRKAA2 | 0.899196968 | 0.310215193 | -1.535367826 | 2.01E-15 | 6.36E-15 |
| ACOT9 | 4.751838195 | 8.191334059 | 0.78561272 | 2.60E-17 | 1.09E-16 |
| NCAPH2 | 11.44509637 | 16.21347269 | 0.502463518 | 7.84E-10 | 1.47E-09 |
| CYP4F22 | 0.09833829 | 0.065175058 | -0.593433297 | 1.47E-06 | 2.24E-06 |
| FADS1 | 0.944752193 | 2.860379066 | 1.598198483 | 8.39E-11 | 1.70E-10 |
| HMGCS2 | 373.6542061 | 80.98152335 | -2.206039075 | 2.00E-18 | 9.99E-18 |
| MECR | 3.711202146 | 5.269878024 | 0.505882984 | 1.17E-11 | 2.60E-11 |
| CPOX | 4.569070488 | 9.307801833 | 1.026539796 | 4.15E-23 | 6.57E-22 |
| HSD17B11 | 102.1275271 | 44.80659641 | -1.188588731 | 4.18E-20 | 2.74E-19 |
| HACD4 | 1.376169151 | 0.670248458 | -1.03788991 | 1.52E-15 | 4.86E-15 |
| NTHL1 | 7.098370049 | 16.34325245 | 1.203135429 | 5.42E-18 | 2.47E-17 |
| MIX23 | 6.875613829 | 10.81858329 | 0.653951165 | 6.47E-09 | 1.14E-08 |
| SDHD | 59.74368463 | 31.75425435 | -0.911836324 | 2.33E-20 | 1.61E-19 |
| HSPH1 | 8.307614976 | 26.58306707 | 1.678001308 | 4.83E-16 | 1.71E-15 |
| MID1IP1 | 10.26777795 | 21.56019147 | 1.070245988 | 1.03E-18 | 5.55E-18 |
| ALDH1B1 | 35.28406366 | 57.64875479 | 0.708272721 | 7.58E-07 | 1.19E-06 |
| PPARD | 25.30029173 | 13.55622242 | -0.900198808 | 2.05E-14 | 6.05E-14 |
| PTGDS | 17.47545895 | 7.314089784 | -1.256580103 | 3.04E-12 | 7.11E-12 |
| HADHA | 117.52819 | 66.99249478 | -0.810935456 | 2.18E-21 | 1.78E-20 |
| ABCC1 | 5.519824512 | 12.51944529 | 1.181476334 | 2.92E-19 | 1.70E-18 |
| PTGR1 | 17.22306585 | 8.579252281 | -1.005418156 | 4.55E-12 | 1.06E-11 |
| FABP2 | 25.21012422 | 3.116325808 | -3.016085248 | 1.05E-22 | 1.23E-21 |
| PHYH | 22.77266751 | 12.02774946 | -0.920936572 | 2.40E-15 | 7.52E-15 |
| CYP2C8 | 0.250929127 | 0.086672858 | -1.533627761 | 3.94E-07 | 6.24E-07 |
| CYP2J2 | 17.40285849 | 11.27903751 | -0.625680332 | 1.30E-07 | 2.08E-07 |
| RXRA | 13.80821793 | 7.34348881 | -0.9109896 | 1.48E-19 | 9.24E-19 |
| THEM4 | 2.163056951 | 3.653187306 | 0.756084075 | 6.45E-13 | 1.62E-12 |
| FABP1 | 1065.426534 | 164.8822206 | -2.691923372 | 5.52E-23 | 7.42E-22 |
| CBR3 | 2.540768617 | 3.954117631 | 0.638090793 | 0.014731923 | 0.017305184 |
| PTGES3 | 82.40899902 | 126.9282764 | 0.623139708 | 6.08E-14 | 1.70E-13 |
| CRYZ | 5.483685951 | 8.368880148 | 0.609888636 | 1.16E-05 | 1.71E-05 |
| CPT1B | 0.273506696 | 0.482723166 | 0.819619909 | 0.000430264 | 0.000581613 |
| CYP8B1 | 0.042909142 | 0.016518347 | -1.377215777 | 2.07E-14 | 6.05E-14 |
| ADH1C | 225.0558468 | 14.21811144 | -3.984481293 | 1.46E-25 | 1.59E-23 |
| SUCLG2 | 92.91314073 | 40.42620951 | -1.200591714 | 6.67E-22 | 6.19E-21 |
| THRSP | 0.533615907 | 0.194939914 | -1.452772159 | 0.005173515 | 0.006325798 |
| ECH1 | 97.77838488 | 60.45010197 | -0.693770802 | 1.36E-15 | 4.42E-15 |
| ELOVL3 | 0.090520017 | 0.215802124 | 1.253400306 | 0.002761546 | 0.003455144 |
| TDO2 | 0.315147181 | 1.344581796 | 2.09305986 | 1.25E-08 | 2.13E-08 |
| ACADS | 86.75642463 | 21.08419266 | -2.040808819 | 2.10E-23 | 4.34E-22 |
| TBXAS1 | 2.731065073 | 5.681220872 | 1.056737303 | 5.10E-07 | 8.02E-07 |
| ACOT11 | 5.599045927 | 3.189845826 | -0.811694318 | 1.14E-15 | 3.79E-15 |
| PTGS1 | 18.20936193 | 2.868671999 | -2.666225444 | 2.62E-23 | 4.80E-22 |
| EPHX1 | 37.51884902 | 22.516778 | -0.736615168 | 4.91E-16 | 1.72E-15 |
| ACSBG2 | 0.023521226 | 0.049508366 | 1.073709054 | 0.044604437 | 0.04999414 |
| ACSF2 | 15.66292327 | 5.917737622 | -1.404235859 | 5.98E-17 | 2.40E-16 |
| ME1 | 3.993067195 | 10.12626125 | 1.342532355 | 1.84E-11 | 4.02E-11 |
| FASN | 15.20311715 | 43.72701424 | 1.524157687 | 4.43E-17 | 1.84E-16 |
| DPEP1 | 18.24079952 | 101.6951359 | 2.479009804 | 1.84E-19 | 1.13E-18 |
| GPX2 | 210.7563688 | 458.4490183 | 1.12118508 | 5.25E-11 | 1.09E-10 |
| AKR1C3 | 29.37900466 | 14.8986968 | -0.979599376 | 2.16E-12 | 5.18E-12 |
| CYP4F3 | 1.430198695 | 3.702165184 | 1.372153675 | 1.92E-08 | 3.21E-08 |
| MCAT | 7.085132488 | 10.59432793 | 0.580425335 | 4.52E-10 | 8.69E-10 |
| ODC1 | 36.86298854 | 87.1467056 | 1.24127309 | 2.43E-18 | 1.15E-17 |
| ALAD | 16.23796829 | 10.19101275 | -0.672073703 | 1.03E-18 | 5.55E-18 |
| CIDEA | 0.331045179 | 0.125782306 | -1.396099132 | 1.65E-24 | 5.65E-23 |
| ELOVL4 | 0.538360651 | 0.224427617 | -1.262322751 | 3.51E-15 | 1.07E-14 |
| CA2 | 644.6842765 | 25.97692151 | -4.633290423 | 4.58E-24 | 1.25E-22 |
| RDH11 | 7.802089659 | 13.02792518 | 0.739674857 | 2.68E-11 | 5.76E-11 |
| AOC3 | 28.93730195 | 5.313263879 | -2.445260145 | 1.78E-18 | 9.23E-18 |
| LDHA | 99.42543732 | 155.0569281 | 0.64111108 | 5.69E-11 | 1.17E-10 |
| ACADM | 18.39971132 | 8.453535441 | -1.122056394 | 1.94E-19 | 1.16E-18 |
| HADHB | 47.10622195 | 26.23544092 | -0.844400592 | 1.39E-22 | 1.50E-21 |
| ADH6 | 4.718287466 | 2.084578279 | -1.17850777 | 6.84E-14 | 1.90E-13 |
| ACADSB | 7.477508293 | 4.72938752 | -0.660904246 | 3.73E-13 | 9.54E-13 |
| ACSL1 | 5.947398902 | 8.494394518 | 0.514252272 | 0.001817696 | 0.002295588 |
| ACAT1 | 23.01784439 | 10.40784464 | -1.1450814 | 8.71E-22 | 7.56E-21 |
| G0S2 | 10.10436729 | 19.39263299 | 0.940529709 | 0.002962281 | 0.003689137 |
| CROT | 6.444056488 | 2.878817598 | -1.162492758 | 5.72E-19 | 3.24E-18 |
| GABARAPL1 | 10.79142527 | 6.615446287 | -0.705975029 | 1.67E-14 | 4.98E-14 |
| NSDHL | 10.61521285 | 23.12394002 | 1.123253935 | 1.10E-19 | 7.07E-19 |
| PTS | 4.316980268 | 6.51168697 | 0.593008849 | 1.66E-12 | 4.03E-12 |
| GPX4 | 59.00556098 | 92.36377143 | 0.646476155 | 2.74E-09 | 4.98E-09 |
| CPT1A | 49.81219268 | 22.44143181 | -1.150334192 | 1.79E-20 | 1.26E-19 |
| FAAH2 | 3.057414624 | 5.833588486 | 0.932071406 | 1.33E-13 | 3.54E-13 |
| GSTZ1 | 3.15667201 | 2.146663169 | -0.55630853 | 4.45E-09 | 7.98E-09 |
| ACSS1 | 6.95236239 | 10.8033278 | 0.635900591 | 0.001624945 | 0.002061841 |
| HACD3 | 9.09761922 | 21.07674924 | 1.212091414 | 2.69E-22 | 2.78E-21 |
| ETFDH | 14.35900598 | 4.353234088 | -1.721796374 | 8.35E-26 | 1.59E-23 |
| ACSM3 | 3.662474222 | 2.511359036 | -0.544350307 | 1.96E-08 | 3.25E-08 |
| HADH | 65.40913634 | 37.45069524 | -0.804499664 | 5.43E-16 | 1.87E-15 |
| ACBD7 | 0.17151476 | 0.805167025 | 2.23095535 | 2.15E-17 | 9.20E-17 |
| SCP2 | 29.92909683 | 15.57547993 | -0.942272126 | 1.25E-22 | 1.40E-21 |
| AUH | 11.07464939 | 7.364725922 | -0.588557284 | 8.30E-12 | 1.88E-11 |
| SLC22A5 | 6.309964229 | 1.863518774 | -1.759602473 | 3.55E-20 | 2.39E-19 |
| H2AZ1 | 64.81058171 | 118.7996215 | 0.874228951 | 5.29E-15 | 1.60E-14 |
| SMS | 28.25992805 | 52.30242135 | 0.888119945 | 2.43E-18 | 1.15E-17 |
| ELOVL5 | 7.748626073 | 17.6163492 | 1.184902541 | 5.57E-11 | 1.15E-10 |
| NUDT7 | 2.796031171 | 1.937614785 | -0.529098665 | 5.62E-10 | 1.06E-09 |
| VNN1 | 1.345739382 | 4.796965857 | 1.833723129 | 0.000370501 | 0.000505913 |
| CYP4B1 | 0.709804391 | 0.10502044 | -2.756751351 | 1.11E-16 | 4.22E-16 |
| MMUT | 17.3914839 | 11.41758498 | -0.607123506 | 1.20E-16 | 4.47E-16 |
| ACO2 | 60.33466049 | 30.38296575 | -0.989724325 | 7.34E-22 | 6.59E-21 |
| UGDH | 59.5753661 | 18.90443658 | -1.65599106 | 8.35E-23 | 1.02E-21 |
| HSD17B12 | 12.13032741 | 18.76122805 | 0.629135774 | 2.83E-09 | 5.11E-09 |
| PPT1 | 24.59272585 | 37.32934381 | 0.602078497 | 2.33E-09 | 4.30E-09 |
| DPEP2 | 1.112139634 | 0.485173394 | -1.196765593 | 4.18E-16 | 1.50E-15 |
| EPHX2 | 26.22799068 | 10.3267473 | -1.344721379 | 5.78E-19 | 3.24E-18 |
| ACOT4 | 2.86497388 | 1.683014924 | -0.767474016 | 1.01E-08 | 1.76E-08 |
| PTGS2 | 1.486429483 | 4.198018819 | 1.497857611 | 0.035423368 | 0.040376635 |
| PTPRG | 2.367322805 | 3.430077298 | 0.534984645 | 4.35E-06 | 6.54E-06 |
| SERINC1 | 68.67286829 | 41.99558715 | -0.709502484 | 2.23E-18 | 1.09E-17 |
| HSD17B10 | 53.19541146 | 102.2871269 | 0.943250877 | 2.83E-16 | 1.03E-15 |
| ACLY | 24.3599339 | 42.57751197 | 0.805581429 | 1.99E-18 | 9.99E-18 |
| TECR | 19.86372093 | 28.25881483 | 0.508565063 | 2.34E-13 | 6.12E-13 |
| ECI2 | 14.66922866 | 6.197732786 | -1.242980553 | 2.16E-21 | 1.78E-20 |
| APEX1 | 53.72487073 | 89.23007567 | 0.731939957 | 7.94E-14 | 2.16E-13 |
| ELOVL6 | 8.885480366 | 5.259735261 | -0.756459587 | 8.17E-12 | 1.86E-11 |
| MIF | 22.02999946 | 56.21578364 | 1.351505791 | 7.71E-16 | 2.62E-15 |
| RETSAT | 97.99502366 | 28.24286426 | -1.794822082 | 6.15E-23 | 7.88E-22 |
| NUDT19 | 6.998136244 | 11.96228952 | 0.773450883 | 2.84E-15 | 8.79E-15 |
| HPGDS | 2.94248561 | 0.371003994 | -2.987528738 | 2.04E-25 | 1.59E-23 |
| ADSL | 7.992861098 | 12.79962242 | 0.67931733 | 1.35E-12 | 3.31E-12 |
| SCD | 19.59543322 | 106.5973575 | 2.443582301 | 3.50E-21 | 2.69E-20 |
| RDH16 | 0.04534584 | 0.248008664 | 2.451348424 | 1.08E-16 | 4.15E-16 |
| SLC25A20 | 29.10013171 | 10.78900039 | -1.431464479 | 3.02E-23 | 5.08E-22 |
| ACOX1 | 34.79789378 | 12.75562507 | -1.447866388 | 1.30E-17 | 5.66E-17 |
| ACSL6 | 0.082178311 | 1.870173839 | 4.508270882 | 4.65E-10 | 8.87E-10 |
| ACSL4 | 4.822209488 | 10.69066619 | 1.148585526 | 2.99E-12 | 7.06E-12 |
| AQP7 | 2.080139902 | 0.574522948 | -1.856244136 | 1.72E-18 | 9.05E-18 |
| IL4I1 | 1.992899627 | 4.113159523 | 1.045377976 | 0.00111448 | 0.001455316 |
| ACADL | 0.050218783 | 0.024989622 | -1.006897975 | 7.03E-13 | 1.75E-12 |
| PTGIS | 4.462056656 | 1.94567787 | -1.197435957 | 1.03E-11 | 2.30E-11 |
| REEP6 | 6.651216456 | 10.87651124 | 0.709525743 | 4.03E-14 | 1.17E-13 |
| HMGCL | 15.42278829 | 9.161020425 | -0.751483403 | 4.69E-17 | 1.91E-16 |
| ACACB | 4.345357171 | 1.378468528 | -1.656408436 | 1.13E-23 | 2.53E-22 |
| ADH1B | 19.01613698 | 1.073188845 | -4.147248332 | 3.07E-25 | 1.65E-23 |
| DECR1 | 31.47676561 | 21.61032838 | -0.54256631 | 1.20E-11 | 2.64E-11 |
| ENO2 | 4.855013 | 7.251706152 | 0.578845308 | 0.019270798 | 0.022344158 |
| LTC4S | 0.023283455 | 0.014214849 | -0.711906361 | 6.96E-05 | 9.85E-05 |
| NBN | 7.705350244 | 11.81876425 | 0.617146757 | 6.64E-08 | 1.09E-07 |
| MGLL | 42.62392488 | 16.467049 | -1.372081407 | 5.46E-23 | 7.42E-22 |
| SLC27A2 | 18.48286795 | 11.37730971 | -0.700029178 | 1.51E-08 | 2.55E-08 |
| HSP90AA1 | 136.8497283 | 238.6525778 | 0.802319349 | 2.38E-13 | 6.16E-13 |
| PCBD1 | 39.6692739 | 69.98937156 | 0.819113863 | 6.57E-18 | 2.90E-17 |
| SLC25A17 | 3.31043761 | 5.38989178 | 0.703234366 | 1.63E-16 | 6.02E-16 |
| SUCLG1 | 50.32588707 | 33.47680901 | -0.588138679 | 1.19E-13 | 3.21E-13 |
| CPT2 | 25.59107615 | 10.07387878 | -1.34502154 | 2.67E-23 | 4.80E-22 |
| ACOT12 | 0.016292568 | 0.007328808 | -1.152563513 | 4.59E-09 | 8.19E-09 |
| TP53INP2 | 55.12963805 | 10.29072996 | -2.421482808 | 2.36E-25 | 1.59E-23 |
| CRAT | 32.53256951 | 17.35802645 | -0.906281847 | 4.24E-14 | 1.21E-13 |
| HSD17B7 | 1.61953118 | 3.462360379 | 1.096179651 | 6.20E-18 | 2.78E-17 |
| ACOT1 | 2.665097756 | 1.76089277 | -0.597881393 | 1.15E-08 | 1.98E-08 |

**SUPPLEMENTARY TABLE 2**

**Eight fatty acid metabolism-related prognostic genes significantly associated with OS of COAD**

| **Gene** | **Multivariate cox regression analysis** | | | | |
| --- | --- | --- | --- | --- | --- |
|  | **Coef** | **HR** | **HR.95L** | **HR.95H** | ***p*-value** |
| CD36 | 1.090824766 | 2.976728164 | 1.322946839 | 6.697858371 | 0.008380483 |
| ENO3 | 2.449937292 | 11.58762006 | 2.126650868 | 63.13821444 | 0.004621968 |
| ACBD4 | 2.623886937 | 13.78921738 | 1.484262009 | 128.1057621 | 0.021042522 |
| SUCLG2 | 2.313619642 | 10.11095654 | 0.88770494 | 115.1637639 | 0.062322155 |
| ELOVL3 | 1.895084335 | 6.653109471 | 1.434626271 | 30.85393494 | 0.015476563 |
| ELOVL6 | -1.093983349 | 0.33487989 | 0.077696714 | 1.443362726 | 0.142200893 |
| ACOX1 | -2.387977395 | 0.091815202 | 0.011153695 | 0.755806185 | 0.026400291 |
| CPT2 | -2.73500507 | 0.064893678 | 0.007167449 | 0.587543653 | 0.014971954 |

**SUPPLEMENTARY TABLE 3**

**Univariate cox regression analysis showing associations between 15 FAMRGs and OS in COAD**

| **Gene** | **Univariate cox regression analysis** | | | |
| --- | --- | --- | --- | --- |
|  | **HR** | **HR.95L** | **HR.95H** | ***p*-value** |
| CD36 | 2.553114671 | 1.142447452 | 5.705640563 | 0.022339155 |
| ENO3 | 19.69938316 | 4.509614747 | 86.05295992 | 7.42E-05 |
| ACBD4 | 12.69239631 | 1.750468703 | 92.03073654 | 0.011941485 |
| MORC2 | 31.91382044 | 2.383672227 | 427.2785172 | 0.008891739 |
| SUCLG2 | 0.187662167 | 0.039232664 | 0.897647137 | 0.036154864 |
| ELOVL3 | 7.171177816 | 1.815112262 | 28.33201689 | 0.004948063 |
| ACOT11 | 0.224871614 | 0.055157127 | 0.916785294 | 0.037421641 |
| ALAD | 23.77398497 | 1.937933292 | 291.6521242 | 0.013240874 |
| CIDEA | 6.66805236 | 1.62210162 | 27.41068853 | 0.008522288 |
| HADH | 0.156076375 | 0.025842738 | 0.942618178 | 0.042932112 |
| ELOVL6 | 0.211795888 | 0.056745623 | 0.790501465 | 0.020898642 |
| ACOX1 | 0.081067479 | 0.01256393 | 0.52307963 | 0.008261722 |
| ACADL | 25.73143244 | 2.153747419 | 307.4207354 | 0.010282768 |
| ENO2 | 3.051910444 | 1.332986369 | 6.987436314 | 0.008289678 |
| CPT2 | 0.055855296 | 0.008871791 | 0.351655512 | 0.002117243 |
